# Estimation of leaf traits from reflectance measurements: comparison between methods based on vegetation indices and several versions of the PROSPECT model

**DOI:** 10.1186/s13007-018-0291-x

**Published:** 2018-03-20

**Authors:** Jingyi Jiang, Alexis Comar, Philippe Burger, Pierre Bancal, Marie Weiss, Frédéric Baret

**Affiliations:** 1 EMMAH UMR 1114, INRA, UAPV, 84914 Avignon, France; 2 HIPHEN, Avignon, France; 3 AGIR UMR 1248, INRA, Toulouse, France; 4 UMR ECOSYS, INRA, Grignon, France

**Keywords:** Chlorophyll content, Carotenoid content, Water content, Dry matter content, Radiative transfer model, Reflectance, Transmittance, Leaf, Wheat, Phenotyping

## Abstract

**Background:**

Leaf biochemical composition corresponds to traits related to the plant state and its functioning. This study puts the emphasis on the main leaf absorbers: chlorophyll a and b ($C_{ab}$), carotenoids ($C_{c}$), water ($C_{w}$) and dry mater ($C_{m}$) contents. Two main approaches were used to estimate [$C_{ab} ,$ $C_{c}$, $C_{w}$, $C_{m}$] in a non-destructive way using spectral measurements. The first one consists in building empirical relationships from experimental datasets using either the raw reflectances or their combination into vegetation indices (VI). The second one relies on the inversion of physically based models of leaf optical properties. Although the first approach is commonly used, the calibration of the empirical relationships is generally conducted over a limited dataset. Consequently, poor predictions may be observed when applying them on cases that are not represented in the training dataset, i.e. when dealing with different species, genotypes or under contrasted environmental conditions. The retrieval performances of the selected VIs were thus compared to the ones of four PROSPECT model versions based on reflectance data acquired at two phenological stages, over six wheat genotypes grown under three different nitrogen fertilizations and two sowing density modalities. Leaf reflectance was measured in the lab with a spectrophotometer equipped with an integrating sphere, the leaf being placed in front of a white Teflon background to increase the sensitivity to leaf biochemical composition. Destructive measurements of [$C_{ab} ,$ $C_{c}$, $C_{w}$, $C_{m}$] were performed concurrently.

**Results:**

The destructive measurements demonstrated that the carotenoid, $C_{c}$, and chlorophyll, $C_{ab}$, contents were strongly correlated (r^2^ = 0.91). The sum of $C_{ab}$ and $C_{c}$, i.e. the total chlorophyllian pigment content, $C_{abc}$, was therefore used in this study. When inverting the PROSPECT model, accounting for the brown pigment content, $C_{bp}$, was necessary when leaves started to senesce. The values of $C_{abc}$ and $C_{w}$ were well estimated (r^2^ = 0.81 and r^2^ = 0.88 respectively) while the dry matter content, $C_{m}$, was poorly estimated (r^2^ = 0.00). Retrieval of $C_{w}$ from PROSPECT versions was only slightly biased, while substantial overestimation of $C_{abc }$ was observed. The ranking between estimated values of $C_{abc}$ and $C_{w}$ from the several PROSPECT versions and that derived using the VIs were similar to the ranking observed over the destructively measured values of $C_{abc}$ and $C_{w}$.

**Conclusions:**

PROSPECT model inversion and empirical VI approach provide similar retrieval performances and are useful methods to estimate leaf biochemical composition from spectral measurements. However, the PROSPECT model inversion gives potential access to additional traits on surface reflectivity and leaf internal structure. This study suggests that non-destructive estimation of leaf chlorophyll and water contents is a relevant method to provide leaf traits with relatively high throughput.

**Electronic supplementary material:**

The online version of this article (10.1186/s13007-018-0291-x) contains supplementary material, which is available to authorized users.

## Background

Plant phenotyping was recognized as one of the major bottleneck in the genetic improvement of crops [1]. It is currently a rapidly growing research domain that follows the continuous technical advances of sensors, robotics and computer systems for data processing. It relies on non-destructive and high-throughput measurements used to assess functional traits repeatedly throughout the growing season [2]. Plant phenotyping is completed at three main scales [3]: (1) the plot scale, i.e. a collection of plants mostly sampled in field conditions, (2) the plant scale generally measured under controlled conditions in the greenhouse, and (3) the organ scale, i.e. an element of the plant (leaf, stem, reproductive or storage organs) that can be sampled either in the field or under controlled conditions. For phenotyping purposes, the leaf biochemical composition provides valuable information on the plant state regarding some key processes such as photosynthesis, respiration and transpiration. The close relationship between chlorophyll and carotenoid pigments and nitrogen status of crops was indeed investigated by several studies [4–10] and depends on crop phenological stages as well on the leaf light environment [11–14]. Variation of the leaf relative water content (water mass per unit leaf mass) is related to the water stress experienced by the plant [15] or indicates the senescence level [16]. Green leaves show generally small deviations of the relative water content to keep the leaf turgescent while being compatible with biochemical processes [17]. The dry matter content corresponds to the leaf mass per area. It is related to photosynthesis and respiration processes [18–20]. It also controls the transformation of the mass of assimilates produced and allocated to the leaf into a leaf area increment within many crop models [21–23].

Chlorophyll, carotenoid, water and dry matter contents show strong and specific absorption features, which impact the leaf reflectance and transmittance spectra [24]. It is therefore possible to estimate the content of these constituents from the measurement of leaf optical properties [25–27]. Indeed, the actual quantity that drives light reflectance and transmittance is the content (mass of constituent per unit leaf area) rather than the concentration (mass of constituent per unit leaf dry mass): the biochemical content governs the effective path length of light through the leaf and controls thus the leaf reflectance and transmittance through scattering and absorption processes.

The estimation of the leaf chlorophyll and carotenoid content from optical measurements [28, 29] became very popular with the rise of precision farming focusing on nitrogen applications [13]. Empirical relationships between leaf water content and leaf optical properties have also been calibrated over experimental datasets and were demonstrated to be efficient [30–34]. Fewer studies reported attempts to estimate dry matter content from reflectance measurements [26, 35, 36]. These studies are generally reporting results obtained over a wide range of contents due either to interspecific differences or to contrasted environmental conditions such as variation in salinity or in the illumination levels in relation to the position of the leaf in the canopy [35, 36]. However, quantifying the differences expected between genotypes grown under similar conditions is more challenging: the differences between genotypes in pigment, water and dry matter contents are generally limited. In these conditions, a significant part of the variation in leaf optical properties is also due to variations in the leaf mesophyll structure, the distribution of pigments in the leaf volume as well as surface features. This affects the relationships between vegetation indices and chlorophyll content while a physically based model of leaf optical properties should allow to explicitly account for these potentially confounding effects. Furthermore, new genotypes grown under given environmental conditions may have characteristics not well represented in the VI-relationship training database, making the biochemical content estimation uncertain. A recent review of models of leaf optical properties [37] distinguishes three main approaches based either on radiative transfer [38–41], on stochastic processes [42, 43], or on ray tracing [44, 45]. PROSPECT is one of the most widely used leaf radiative transfer models [24, 41, 46, 47]. It has been successfully applied to retrieve leaf biochemical composition from reflectance and/or transmittance measurements [26, 46, 48, 49]. Several versions of the PROSPECT model are available. They mostly differ by the increasing detail in the pigments used and the associated values of the specific absorption coefficients, water and dry matter, as well as by the value of the refractive index controlling the scattering processes in the leaf.

The objective of this study was to evaluate the performances of the several versions of the PROSPECT model to estimate leaf chlorophyll, $C_{ab}$, carotenoid, $C_{c}$, water, $C_{w}$, and dry matter, $C_{m}$, contents from leaf reflectance measurements in the context of phenotyping experiments. Performances were compared to those obtained using empirical relationships with vegetation indices. The study is based on an experiment conducted over six wheat cultivars grown under several nitrogen levels and sowing densities. Leaf reflectance spectra in the 450–2250 nm domain were acquired at two growing stages, concurrently with destructive measurements of chlorophyll, carotenoid, water and dry matter contents. Attention was paid both to the accuracy and precision of the biochemical content estimates as well as to the ranking capacity necessary to identify differences between genotypes.

## Methods

### The biological material

The experiment took place near Toulouse at the INRA centre “Auzeville Tolosane” (43°33′N, 1°28′E) in France over a site presenting deep and homogenous soil conditions. The wheat plants from which the leaves were collected were grown in field conditions described in [2]. The crop was sown in October 2011 and harvested in June 2012. Three factors were taken into account in the experimental design which resulted into 36 modalities: six cultivars (four winter wheat: Apache, Caphorn, Soissons and Hysun (hybrid); two durum wheat: Isildur and Biensur), two sowing densities and three nitrogen levels.

### The measurements

Leaves were collected in April 2012 at the “two nodes” stage and in June 2012 during grain filling. All the 36 modalities were sampled in April, while only 26 of them were collected in June. For each of the resulting 62 samples, six top leaves were randomly collected. Three of them were used for the destructive measurements of dry matter and water content and the remaining three for destructive measurements of chlorophyll and carotenoid. Reflectance measurements were conducted for each of the six leaves used for destructive measurements. All data for destructive and spectral measurements are provided in Additional file 1.

#### Destructive measurements

The area ($S$) of each leaf was first measured by scanning each sample and processing the resulting image with the SCANAREA software [40]. Then, the three leaves used for the destructive measurements of $C_{m}$ and $C_{w}$ were weighed before ($M_{fresh}$), and after ($M_{dry}$) drying them out at 80 °C in an oven during 2 days. The dry matter ($C_{m}$ in mg/cm^2^) and water contents ($C_{w}$ in mg/cm^2^) were then computed using the following equations:

1 $$C_{m} = \frac{{M_{dry} }}{S}$$

2 $$C_{w} = \frac{{M_{fresh} - M_{dry} }}{S}$$

The three leaves used for $C_{ab}$ and $C_{c}$ leaves were lyophilized and stored in the dark at − 20 °C after measuring their area. The mass of Chlorophyll a and b and carotenoid were then estimated according to [50] by extracting the pigments in acetone and measuring the optical density of the solution. The corresponding content was computed using the measured area of each leaf.

#### Spectral measurements

**Fig. 1:**
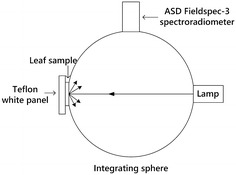
The experimental setup for leaf reflectance measurement with Teflon white panel

The optical properties of the 372 leaves were acquired using an ASD Fieldspec-3 spectroradiometer (Analytical Spectral Devices Inc., Boulder, Colorado, USA) equipped with an integrating sphere Li-Cor 1800-12 (LI-COR Inc., Lincoln, NE). Data were sampled at intervals of 1.4 nm (350–1050 nm) and 2 nm (1000–2500 nm) with a spectral resolution of 3 nm for the region 350–1000 nm and 10 nm for the region 1000–2500 nm [51]. The direction of the incoming light was almost normal to the leaf sample while the bare fiber of the spectroradiometer viewed the integrating sphere wall under a 25° field of view (Fig. 1). The original Li-Cor lamp system of the integrating sphere was replaced by a lamp connected to a stabilized power supply. The original infrared filter was removed to increase the light available in this domain where the spectrophotometer has a lower sensitivity than in the shorter wavelengths. A Teflon white panel was used as the background of the leaf as proposed by [49] to increase the optical path in the leaf, thus enhancing the absorption features. Another Teflon white panel was used as a secondary reference to compute the directional-hemispherical reflectance factor (DHRF) of the leaf-white background system. The absolute $DHRF_{ref}$ of the secondary Teflon white reference was calibrated against a spectralon primary reference panel [52].

Three spectrophotometer measurements were completed for each of the six leaves sampled per date, cultivar and modality. The average ($S_{leaf} \left( \lambda \right)$) of the resulting 18 individual spectra was computed and then transformed into the corresponding DHRF ($DHRF_{leaf} \left( \lambda \right)$) according to Eq. (3):

3 $$DHRF_{leaf} \left( \lambda \right) = \frac{{2 S_{leaf} \left( \lambda \right)}}{{\left( {S_{ref\_bef} \left( \lambda \right) + S_{ref\_aft} \left( \lambda \right)} \right)}}DHRF_{ref} \left( \lambda \right)$$

where $S_{ref\_bef} \left( \lambda \right)$ and $S_{ref\_aft} \left( \lambda \right)$ are the spectra of the secondary Teflon reference completed before and after the series of the 18 leaf spectrophotometer measurements. The reflectance of the white background was measured systematically just after the $S_{ref\_aft} \left( \lambda \right)$ measurements to account for possible changes of its properties due to the contact with the leaf.

### The vegetation indices

A vegetation index is a combination of spectral bands that captures some absorption characteristics of a given biochemical content. Several of them have been proposed in the literature, mainly to assess water [33], and chlorophyll and carotenoid contents [48, 53, 54]. However, their associated performances are still a matter of discussion when the calibration and validation datasets differ in acquisition conditions, crop state and/or soil background [55, 56]. Two VIs (Dx4 and Clre) were selected among the most popular ones for chlorophyll content estimates (Table 1): Dx4 was developed for the Dualex Scientific+™ instrument (Force-A, Orsay, France) to estimate chlorophyll content from the transmittance in the red-edge ($T_{710}$) and the near infrared ($T_{850}$) [29]. CIre is the ratio between the reflectance in the near infrared ($R_{760 - 800}$) and the red-edge ($R_{690 - 710}$) [28, 57]. For water content, two popular indices were selected: SRw [31] is the ratio between reflectance in the short wave infrared ($R_{1300}$; $R_{1450}$) and NDw [27] is a normalized difference of bands in the short wave infrared ($R_{1062} , R_{1393}$). Since all the selected VIs are designed to enhance the absorption features of chlorophyllian pigments or water for leaf transmittance (Dx4) or reflectance over a black background (other VIs), they are also expected to work similarly for leaf optical properties measured over a white background. Simple linear functions were considered to empirically relate the biochemical contents and Dx4, CIre and SRw. A second order polynomial function was used to relate NDw and $C_{w}$. A leave-one-out method was used to quantify the performances of the empirical calibration using the r^2^ (squared Pearson correlation coefficient) and RMSE (root mean square error) between the estimated and measured biochemical contents.

**Table 1:** Definition of the selected vegetation indices

Variables	**VIs**	Formula	References
$C_{\text{abc}}$	Dx4	$\frac{{T_{850} }}{{T_{710} }} - 1$	[29]
	Clre	$\frac{{R_{760 - 800} }}{{R_{690 - 710} }} - 1$	[28, 57]
$C_{\text{w}}$	SRw	$\frac{{R_{1300} }}{{R_{1450} }}$	[31]
	NDw	$\frac{{R_{1062} - R_{1393} }}{{R_{ 1062} + R_{1393} }}$	[27]

### Inversion of the PROSPECT model

#### PROSPECT versions

The PROSPECT model [41] extended to multiple layers (plates) the (single) plate model from Allen [58] using the Stokes system of equations [59]. The mesophyll structure parameter, $N$, characterizes the number of homogenous elementary layers that constitute the leaf. Each elementary layer is described by the refractive index of the leaf material, $n,$ and by an absorption coefficient computed as the sum of the specific absorption coefficients of each constituent weighted by their corresponding content. Several versions of the PROSPECT model have been proposed in the literature. They differ mainly by the specific absorption coefficients and refractive index. The original version was first updated based on a dataset of 58 leaves representing a broad range of species over which the specific absorption coefficients were recalibrated [26]. This resulted into PROSPECT version 3 (P3) [24, 41]. More recently, new values of the specific absorption coefficients and refractive index were proposed by [46] based on a larger set of leaf reflectance and transmittance measurements. It resulted into PROSPECT version 4 (P4) where chlorophyll and carotenoids were pooled together, and PROSPECT version 5 (P5) where chlorophyll and carotenoids were described separately. Finally, PROSPECT-D was proposed by [60], where anthocyanins were described explicitly in addition to chlorophyll a and b and carotenoids. Besides, the refractive index was also recalibrated. Finally, the contribution of the brown pigment content ($C_{bp}$) to leaf absorption can be added to each of the 4 PROSPECT versions, leading to P3b, P4b, P5b and PDb versions (Table 2). Brown pigments correspond to polyphenols that appear during leaf senescence [46].

**Table 2:** Description of the different PROSPECT model versions considered in this study

Version name	PROSPECT 3		PROSPECT 4		PROSPECT 5		PROSPECT D	
Chlorophyllian pigment separation	$C_{abc}$		$C_{abc}$		$C_{ab}$ and $C_{c}$		$C_{ab}$, $C_{c}$ and $C_{Anth}$	
References	[26]		[46]		[46]		[60]	
Brown pigments	$C_{bp} = 0$	$C_{bp}$	$C_{bp} = 0$	$C_{bp}$	$C_{bp} = 0$	$C_{bp}$	$C_{bp} = 0$	$C_{bp}$
Abbreviated name	P3	P3b	P4	P4b	P5	P5b	PD	PDb

#### Adaptation of PROSPECT to the measurement configuration

**Fig. 2:**
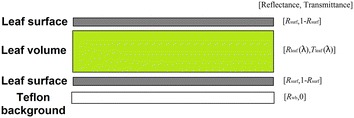
Leaf model to measure reflectance over a white background: the reflectance and transmittance values of each layer are indicated.$R_{surf}$ is the surface reflectivity for both upper and lower leaf surfaces (independent of wavelength), $R_{leaf} \left( \lambda \right)$ and $T_{leaf} \left( \lambda \right)$ are the leaf reflectance and transmittance simulated by PROSPECT, assuming no reflectivity at the top and the bottom of the leaf volume. $R_{wb}$ is the reflectance of the Teflon white background. All the reflectance and transmittance terms are bi-hemispherical except the upper and lower leaf surface reflectivity is directional-hemispherical

The reflectance measurements were achieved with the leaf placed over a white Teflon background to enhance the sensitivity to the leaf biochemical composition by increasing the optical path in the leaf [49]. PROSPECT simulates the directional hemispherical reflectance ($R_{leaf}$) and transmittance ($T_{leaf}$) of the leaf from the knowledge of the chlorophyll, carotenoid, water and dry matter contents, as well as brown pigments and the mesophyll structure parameter, $N$ [41, 46]. In this study, the computation of the surface reflectivity was approximated by using the parameter $R_{surf}$ conversely to the original PROSPECT version where the ‘α’ solid angle was used to mimic the leaf surface roughness. This allows to get a wider range of variability of surface reflectivity in agreement with observations [61]. $R_{surf}$ was assumed to be independent from wavelength since the refractive index is very little spectrally dependent in the 350–2500 nm domain [61, 62]. Because wheat presents only small differences between the upper and lower surface features, $R_{surf}$ was assumed to be the same for both faces. Indeed, the possible small differences between the two faces have a marginal impact on leaf characteristics estimates since the value of the illuminated face will mainly control the optical properties of the system. Figure 2 showed the representation of the system of layers used to compute leaf reflectance when the leaf was placed over the white Teflon background. The leaf volume layer was characterized by the reflectance and transmittance simulated by PROSPECT assuming no reflectivity at the top and the bottom, while the leaf upper and lower epidermis layers were characterized by $R_{surf}$ with no absorption.

The system described in Fig. 2 was solved in three steps. First the reflectance of the lower leaf surface over the white Teflon background, $R_{surf}^{wb}$, was computed as:

4 $$R_{surf}^{wb} \left( \lambda \right) = R_{surf} + \frac{{R_{wb} \left( \lambda \right)\left( {1 - R_{surf} } \right)^{2} }}{{1 - R_{surf} R_{wb} \left( \lambda \right)}}$$

where $R_{surf}$ is the reflectivity of the lower surface, assuming that the transmissivity of the interface is $1 - R_{surf}$ and there is no absorption at the leaf surface. $R_{wb} \left( \lambda \right)$ is the hemispherical reflectance of the Teflon white background. The reflectance at the bottom of the upper epidermis, $R_{bue}^{wb} \left( \lambda \right)$, was then computed as:

5 $$R_{bue}^{wb} \left( \lambda \right) = R_{leaf} \left( \lambda \right) + \frac{{R_{surf}^{wb} \left( \lambda \right)T_{leaf} \left( \lambda \right)^{2} }}{{1 - R_{leaf} \left( \lambda \right) R_{surf}^{wb} \left( \lambda \right)}}$$

where $R_{leaf} \left( \lambda \right)$ is the leaf volume reflectance computed from the PROSPECT model for which the reflectivity of the surface of the leaf volume is set to 0; $T_{leaf} \left( \lambda \right)$ is the corresponding leaf volume transmittance. Note that Eq. (5) assumes that the properties of the leaf are the same on both faces and that the directional hemispherical reflectance and transmittance are equal to the bi-hemispherical corresponding quantities. Then, the reflectance of the leaf over the white background was computed using the upper surface reflectivity which was assumed to be identical to the lower surface:

6 $$R_{leaf}^{wb} \left( \lambda \right) = R_{surf} \left( \lambda \right) + \frac{{R_{bue}^{wb} \left( \lambda \right)\left( {1 - R_{surf} \left( \lambda \right)} \right)^{2} }}{{\left( {1 - R_{surf} \left( \lambda \right)R_{bue}^{wb} \left( \lambda \right)} \right)}}$$

Finally, since the incident light on the leaf may directly illuminate the white background in case of small leaves, an additional parameter, $f_{wb}$, was introduced to describe this situation. $f_{wb}$ is the fraction of white Teflon background illuminated directly by the light source. The corresponding reflectance of the system was finally written as:

7 $$R\left( \lambda \right) = R_{wb} \left( \lambda \right).f_{wb} + \left( {1 - f_{wb} } \right).R_{leaf}^{wb} \left( \lambda \right)$$

#### Fitting the white background PROSPECT model variables

An iterative minimization of the cost function, $J\left( V \right)$ (Eq. 8), was applied to estimate the model variables, $V$, where $V$ = [$C_{abc}$, $C_{w}$, $C_{m}$, $N$, *R*_*surf*_, $f_{wb}$] for P3 and P4, $V$ = [$C_{ab}$, $C_{c}$, $C_{w}$, $C_{m}$, $N, R_{surf}$, $f_{wb}$] for P5 and $V$ = [$C_{ab}$, $C_{c}$, $C_{Anth} , C_{w}$, $C_{m}$, $N, R_{surf}$, $f_{wb}$] for PD. The brown pigments $C_{bp}$ were also considered as an additional variable for each of the four models (P3b, P4b, P5b, PDb).

The cost function $J\left( V \right)$ computed the distance between the PROSPECT simulated reflectance spectrum and the actual measurements over the 18 acquisitions performed on each date, cultivar and modality:

8 $$J\left( V \right) = \sqrt {\frac{1}{1800}\mathop \sum \limits_{\lambda = 400}^{\lambda = 2200} \left( {R_{prospect}^{wb*} \left( \lambda \right) - R_{leaf}^{wb} \left( \lambda \right)} \right)^{2} }$$

The original 300–2500 nm spectral range of the ASD spectroradiometer was restricted to the 400–2200 nm domain because (1) the PROSPECT model was calibrated only for wavelengths higher than 400 nm and (2) the signal was dominated by noise for wavelengths longer than 2200 nm. Furthermore, the 400–2200 nm spectral domain contains a significant part of all the spectral features of the biochemical components considered in this study.

The interior point minimization algorithm [63] was used to minimize $J\left( P \right)$ by keeping the variables within their bounds (Table 3). Three initial guesses (Table 3) were used to avoid the algorithm to be trapped in a local minimum. The estimated biochemical contents were then computed as the mean value over the three optimization results. Fortunately, in most situations the three initial guesses were providing almost the same solution.

**Table 3:** Initial guesses and bounding limits required to perform the fitting of the PROSPECT models

Variables	$C_{c}$ (µg/cm^2^)	$C_{ab}$ (µg/cm^2^)	$C_{abc}$ (µg/cm^2^)	$C_{Anth}$ (µg/cm^2^)	$C_{m}$ (mg/cm^2^)	$C_{w}$ (mg/cm^2^)	$C_{bp}$	$N$	$R_{surf}$	$f_{wb}$
*Initial guess*										
1	10	50	60	5	12	5	0.01	1.4	0.05	0.01
2	5	20	20	1	8	1.5	0.2	2	0.1	0.2
3	50	80	90	10	40	18	0.001	1.1	0.01	0.1
*Bounds*										
Min	0	0	0	0	1	1	0	1.01	0	0.0
Max	80	140	140	20	50	30	1	3.5	0.5	1.0

List of the three initial guesses and bounding limits used to minimize the cost function for each variable. Min and Max are the minimum and maximum bounding values of each variable

## Results

### Relationships between biochemical contents

The relationships between $C_{ab}$, $C_{c}$, $C_{w}$ and $C_{m}$ were first investigated over the destructive measurements which were considered as the reference. Note that $C_{bp}$ was not measured since polyphenols are difficult to extract.

**Fig. 3:**
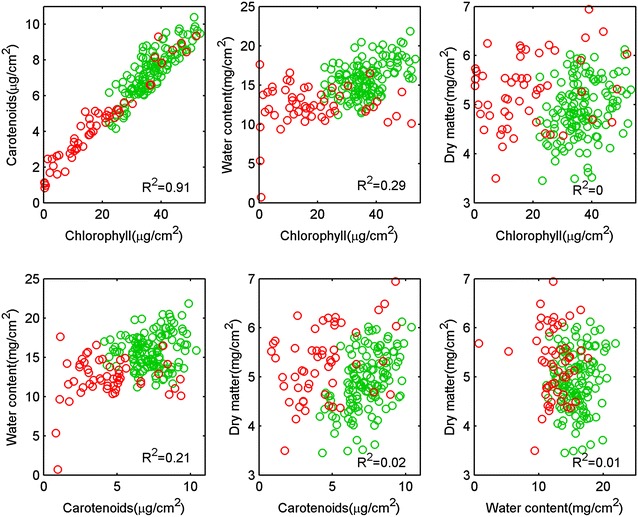
Relationships between the four biochemical leaf traits from destructive measurements. Green and red points correspond to measurements achieved at two nodes (April) and grain filling (June) stages respectively. The squared Pearson correlation coefficient (r^2^) of each relationship is indicated

The results showed that dry matter content was independent from the content of the other constituents with r^2^ lower than 0.02 (Fig. 3). Chlorophyll and, in a lesser extent, carotenoid contents were correlated to water content (r^2^ larger than 0.2 significant at α = 5%) since a loss of water is concomitant with a loss of chlorophyll and carotenoid pigments for the senescing leaves (Fig. 3). The strongest correlation was observed between chlorophyll and carotenoid pigments (r^2^ = 0.91 with a ratio of $C_{ab} /C_{c} 5$, when the offset is neglected Fig. 3), which was consistent with the results from [54] and [41]. However, while these studies found an offset of 5 µg/cm^2^ in this relationship over a large range of species, we observed a lower offset for the carotenoid content (≈1 µg/cm^2^) when all the chlorophyll had disappeared. Considering this strong relationship between chlorophyll and carotenoid contents, we did not consider them separately in the following of the study.

### PROSPECT spectra simulation performances

**Fig. 4:**
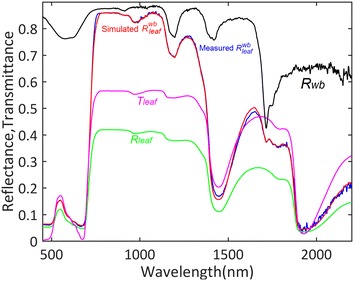
Example of a measured leaf spectrum (red) and a corresponding PROSPECT simulation (blue). The reflectance (*R*_*leaf*_ and *T*_*leaf*_) and transmittance of the leaf volume are shown in green and magenta respectively. The reflectance computed at the top of the leaf volume (*R*_*wb*_) considering measurements over a Teflon white background is shown in black

**Fig. 5:**
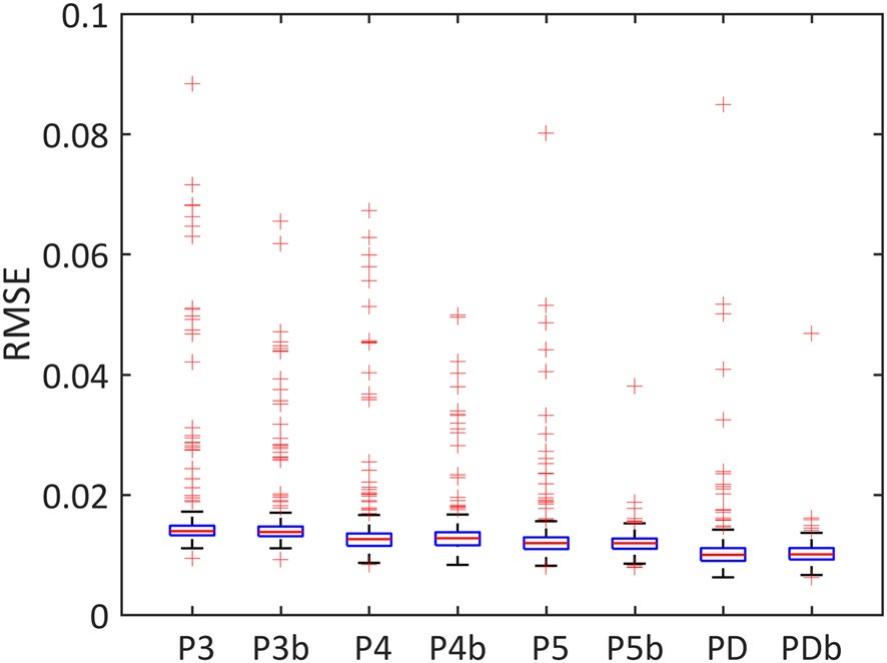
Box plot of RMSE between the measured and simulated reflectance using the 8 PROSPECT versions (P3:PROSPECT 3, P3b: PROSPECT 3 considering the brown pigment content, P4:PROSPECT 4, P4b: PROSPECT 4 considering the brown pigment content, P5:PROSPECT 5, P5b: PROSPECT 5 considering the brown pigment content, PD: PROSPECT D, PDb: PROSPECT D considering the brown pigment content)

The performances of the inversion were first evaluated by considering the agreement between the simulated and the measured reflectance spectra. Figure 4 shows an example of a measured and simulated leaf reflectance spectrum, as well as the several terms used in Eqs. (4–7). The reflectance was simulated using the estimated values of the variables $V$ after minimizing the cost function $J\left( V \right)$ (Eq. 8). This result showed that the reflectance spectra simulated using the retrieved PROSPECT model variables closely matched the measurements. Indeed, when considering the whole dataset, the average RMSE between the measured and estimated spectra over all the samples and the different PROSPECT versions was 0.013 (Fig. 5). PD and PDb provided the lowest RMSE. The observed outliers corresponded to senescent leaves for which absorption features cannot be properly modeled with the present PROSPECT model versions.

### Performances for biochemical composition estimation

**Fig. 6:**
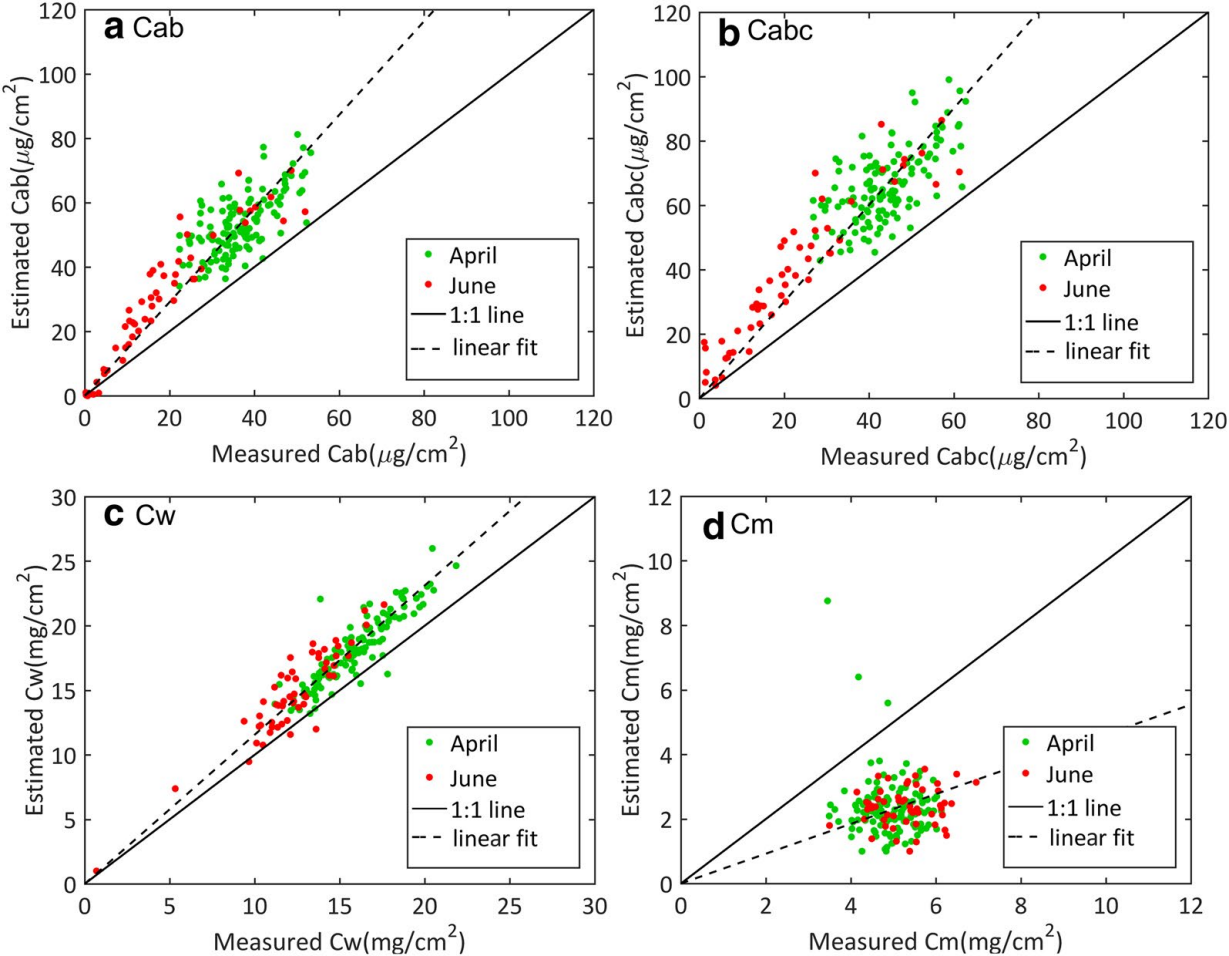
Scatter plots between measured and estimated biochemical contents from PROSPECT PDb (PROSPECT D considering the brown pigment content). The solid line corresponds to the 1:1 line. **a** Chlorophyll and carotenoid content ($C_{\text{abc}}$); **b** chlorophyll content ($C_{\text{ab}}$); **c** water content ($C_{\text{w}}$); **d** dry matter content ($C_{m}$). The dashed line is the best linear fit corrected from the offset. Green and red points correspond to measurements achieved at two nodes (April) and grain filling (June) stages respectively

The values of the retrieved $C_{ab}$, C_c_, *C*_*abc*_, C_w_ and C_m_ were compared to the destructive measurements. Results (Table 4) showed that when combining these pigments into chlorophyllian pigments, $C_{abc}$ estimates were strongly correlated with the destructive measurements for all the PROSPECT versions (r^2^ between 0.59 and 0.79). The addition of brown pigments (P3b, P4b, P5b and PDb) provided more accurate estimation of $C_{abc}$ (r^2^ between 0.79 and 0.81), particularly for the June measurements after the beginning of the senescence (Fig. 6). When correcting the chlorophyll systematic overestimation by a linear fit (Fig. A, dashed line), the RMSE values varied from 6 to 9 µg/cm^2^. However, part of the scattering might also be attributed to uncertainties in the destructive measurements of chlorophyllian pigments used as a reference, estimated to be around 10%, i.e. 3 µg/cm^2^.

**Table 4:** Performances of the inversion process over the 372 sampled leaves

Variables	Metrics	P3	P3b	P4	P4b	P5	P5b	PD	PDb
C_abc_ (µg/cm^2^)	r^2^	0.65	*0.81*	0.59	0.79	0.63	0.80	0.79	0.80
	RMSE	30.92	27.66	19.90	*10.93*	25.85	19.21	25.33	22.72
	RMSE Corr	8.70	*6.51*	9.88	6.67	8.94	6.66	6.83	6.67
	Slope	1.67	1.63	1.35	*1.18*	1.54	1.41	1.57	1.50
	Bias	− 27.70	− 25.09	− 14.99	*−* *8.11*	− 23.29	− 17.56	− 23.22	− 20.69
$C_{ab}$ (µg/cm^2^)	r^2^	–	–	–	–	0.81	*0.82*	0.81	*0.82*
	RMSE					24.60	18.71	19.39	*17.29*
	RMSE Corr					5.60	*5.29*	5.52	5.45
	Slope					1.67	1.50	1.52	*1.45*
	Bias					− 22.38	− 16.78	− 17.52	**−** *15.39*
$C_{c}$ (µg/cm^2^)	r^2^					0.16	0.04	*0.48*	0.45
	RMSE					9.00	*3.91*	6.18	5.75
	RMSE Corr	–	–	–	–	5.63	3.42	*1.56*	1.58
	slope					0.88	*0.99*	1.80	1.74
	Bias					− 0.91	*−* *0.78*	− 5.70	− 5.30
$C_{w}$ (mg/cm^2^)	r^2^	*0.88*	0.87	0.85	0.85	0.86	0.86	0.85	0.85
	RMSE	*2.35*	2.87	3.75	3.91	3.65	3.79	2.47	2.67
	RMSE Corr	1.06	1.13	1.06	1.07	*1.03*	1.04	1.08	1.09
	Slope	*1.13*	1.17	1.23	1.24	1.23	1.24	1.14	1.16
	Bias	*−* *1.96*	− 2.47	− 3.54	− 3.68	− 3.48	− 3.59	− 2.14	− 2.34
$C_{m}$ (mg/cm^2^)	r^2^	0.00	0.00	0.00	0.00	0.00	0.00	0.00	0.00
	RMSE	*2.45*	2.82	2.82	3.07	2.83	3.06	2.59	2.87
	RMSE Corr	*1.67*	1.80	1.80	1.90	1.80	1.89	1.75	1.84
	slope	*0.56*	0.47	0.47	0.42	0.47	0.42	0.53	0.46
	Bias	*2.18*	2.60	2.61	2.88	2.62	2.88	2.33	2.65
$N$	Mean	1.45	1.47	1.64	1.63	1.64	1.62	1.43	1.41
$R_{surf}$	Mean	0.05	0.05	0.01	0.01	0.01	0.01	0.01	0.01
$f_{wb}$	Mean	0.01	0.02	0.07	0.07	0.07	0.07	0.04	0.04

The estimation performances of $C_{abc}$, $C_{ab}$, $C_{c}$, $C_{w}$ and $C_{dm}$ were quantified using the squared Pearson correlation coefficient (r^2^) and the RMSE computed between the measured and estimated biochemical contents over the 186 available data. The RMSE Corr was computed when correcting for possible systematic deviations using a linear model characterized by a slope as observed in Fig. 6. Bias value was the difference between the mean measured and mean estimated biochemical contents. The numbers in italic indicate the best result for each biochemical content and model version

Leaf water content was very well estimated regardless of the PROSPECT version (Table 4). However, a small bias was systematically observed (1.13 ≤ slope ≤ 1.24). The estimation of the dry matter content showed very poor performances, with a r^2^ = 0.00, a significant bias (between 2.18 and 2.88 mg/cm^2^) and RMSE values after bias correction around 1.8 mg/cm^2^.

The mean value of the retrieved N parameter (mesophyll structure) differed according to the four PROSPECT versions: N < 1.5 for P3, P3b, PD and PDb while N > 1.6 for P4, P4b, P5 and P5b. This behavior was partly linked to some compensation effects between $R_{surf}$ and f_wb_ during the model inversion process. The surface component of the leaf reflectance, $R_{surf}$, also varied between the PROSPECT versions. It was found to be 0.05 with P3 and P3b which was in better agreement with the literature [2, 64], as compared to the other PROSPECT versions ($R_{surf}$ = 0.01). Estimates of $f_{wb}$ using P4, P4b, P5, and P5b, were higher ($f_{wb}$ ≈ 0.07) than for P3, P3b, PD and PDb ($f_{wb}$ ≈ 0.03).

### Comparison between $C_{\text{abc}}$ and $C_{\text{w}}$ estimates from PROSPECT and vegetation indices

The comparison was first based on the Spearman correlation coefficient that offered the advantage to be independent from possible bias and little sensitive to the non-linearity between the biochemical contents and the VIs considered in this study. The Spearman correlation coefficient quantifies the consistency of the ranking between the biochemical content measurements used as reference and those estimated from non-destructive techniques. The ranking capacity of phenotyping techniques, i.e. the relative values of traits rather than their absolute values, is indeed probably the first property required by the breeders.

The total chlorophyllian pigment content was here considered since it was difficult to estimate independently the chlorophyll a and b from the carotenoids (Table 5). Furthermore, $C_{\text{ab}}$ and $C_{\text{c}}$ were strongly correlated (Fig. 3). The PROSPECT versions using the brown pigments were considered here because of their better performances.

**Table 5:** Comparison between destructive measurements of $C_{\text{abc}}$ and $C_{\text{w}}$ and PROSPECT or vegetation indices estimates

Variables	Metrics	PROSPECT				VIs			
		P3b	P4b	P5b	PDb	Dx4	CIre	SRw	NDw
$C_{\text{abc}}$ (µg/cm^2^)	ρ	0.81	0.80	*0.82*	0.80	0.80	0.78	–	–
	*r* ^*2*^	*0.81*	0.79	0.80	0.80	0.80	0.77	–	–
	RMSE Corr	*6.54*	6.72	6.70	6.71	6.63	7.08	–	–
$C_{\text{w}}$ (mg/cm^2^)	ρ	0.93	0.93	0.92	0.91	–	–	0.89	*0.94*
	*r* ^*2*^	0.87	0.85	0.86	0.85	–	–	0.80	*0.88*
	RMSE Corr	1.14	1.08	*1.05*	1.11	–	–	1.28	1.29

The estimation performances from the four PROSPECT versions (including brown pigments) and vegetation indices against destructive measurements: spearman correlation coefficient (ρ), squared Pearson correlation coefficient (r^2^) and RMSE Corr as provided in Table 4. RMSE Corr for VIs was computed from the fitted empirical model between the biochemical contents and the VIs: linear functions for Dx4, CIre and SRw, a second order polynomial function for NDw. The numbers in italic indicate the best result for each biochemical content

Results (Table 5) showed that, after bias correction, the performances of $C_{\text{abc}}$ estimation were good for all the versions of PROSPECT and similar to the ones of Dx4. They were slightly degraded for CIre (ρ = 0.78; RMSE Corr = 7.08) as compared to PROSPECT and Dx4 estimates (ρ > 0.8; RMSE Corr = 6.63).

Performances for water content estimation were very good, especially when considering the Spearman correlation coefficient, which was higher than for $C_{\text{abc}}$. The four versions of the PROSPECT model provided similar results after bias correction (Table 5). However, NDw slightly improved the estimation of water content both for ρ and RMSE.

## Discussion

### Accuracy of the PROSPECT versions to simulate reflectance spectra

The performances (RMSE) in terms of the full spectrum reconstruction were decreasing from the first (P3) to the last (PD) version of PROSPECT (Fig. 5). These results did not match the model performances for biochemical content estimation (Table 4) because of three main reasons: (1) there were possible compensations between the several specific absorption coefficients during the PROSPECT calibration process. (2) A bias in the specific absorption coefficient results in a bias in the biochemical content estimates. (3) there might also be compensations between some parameter estimates during the PROSPECT model inversion implemented in this study.

**Fig. 7:**
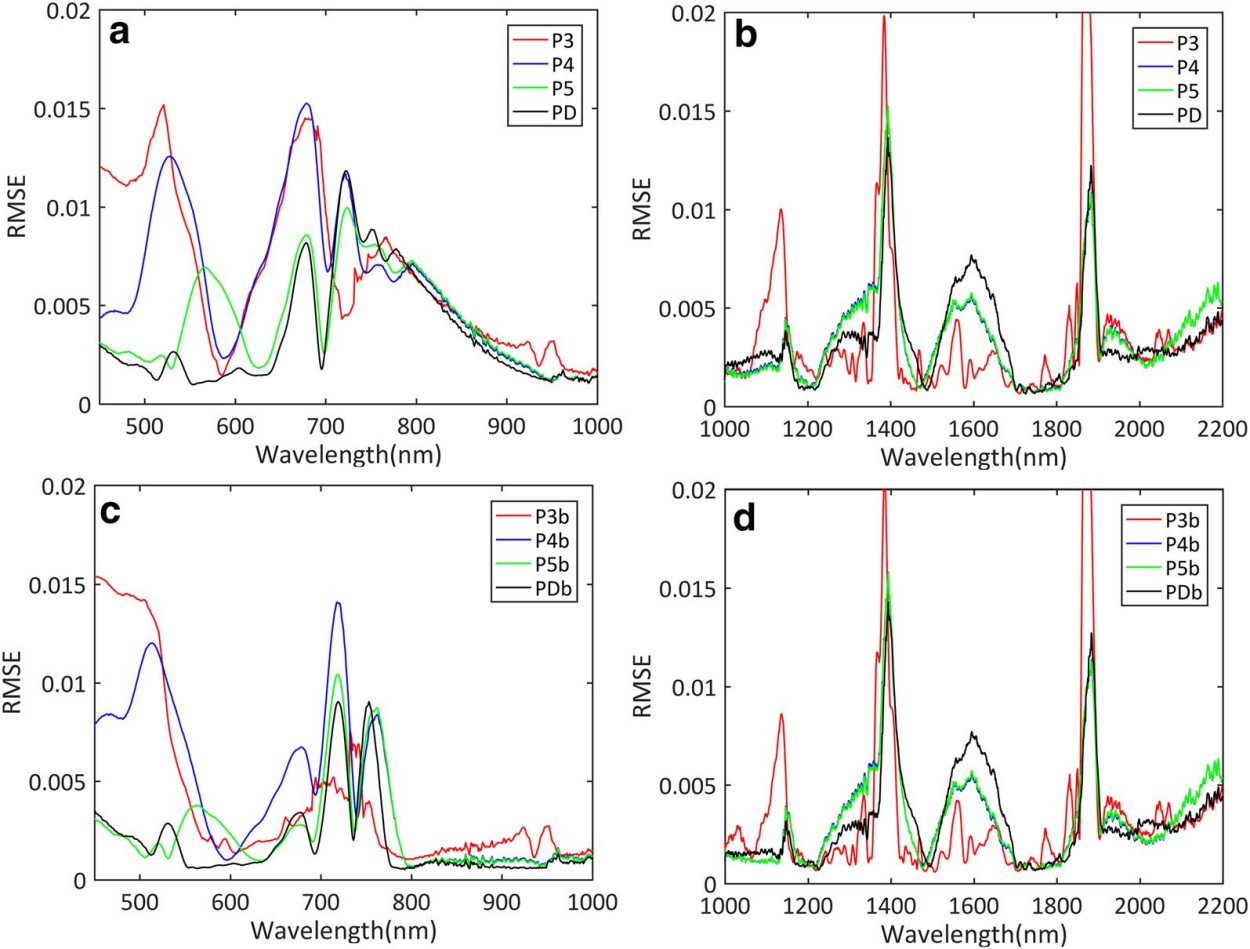
Spectral variation of RMSE between measured and PROSPECT-simulated reflectance. **a** RMSE for the 400–1000 nm domain without brown pigments; **b** RMSE for the 1000–2200 nm domain without brown pigments; **c** RMSE for the 400–1000 nm domain with brown pigments; **d** RMSE for the 1000–2200 nm domain with brown pigments. Different versions of PROSPECT are presented

The inclusion of the brown pigments helped decreasing the number of outliers for all the model versions (Fig. 5), particularly for the June measurements when senescence was observed (results not shown). The spectral variations of the RMSE between measured and simulated reflectance clearly showed the advantage of including the brown pigments to get more accurate and precise reflectance simulations in the 400–1000 nm domain (Fig. 7a, b). Between 1000 nm and 2200 nm (Fig. 7b, d), the effect of the brown pigments was negligible as expected since they do not absorb in these longer wavelengths.

Closer inspection (Fig. 7c) showed that P3b and P4b versions that did not account for the carotenoids showed larger RMSE values in the 400–570 nm domain. In the red edge (700–780 nm), all the PROSPECT versions showed artefacts as compared to the measurements, while the RMSE was much lower for P3b than for the other versions. The separation of anthocyanin from chlorophyllian pigments in PDb further decreased the RMSE between 500 to 600 nm where anthocyanin absorbs light. PDb that describes the biochemical content of more pigments than the other versions showed therefore the best agreement with the measured reflectance spectra.

In the 1000–2200 nm domain, P3 and P3b showed significant RMSE peaks on the lower wavelength shoulders of the main water absorption features at 1150, 1400 and 1900 nm although it performed best at 1300 and 1600 nm. P4, P4b, P5 and P5b showed similar RMSE peaks around the water absorption features.

### Comparison between PROSPECT versions for $C_{abc}$, $C_{w}$ and $C_{m}$ estimates

Taking into account the presence of brown pigments significantly improved the performances of all the PROSPECT model versions to estimate $C_{\text{ab}}$ and $C_{\text{abc}}$, resulting in r^2^ values between 0.79 and 0.81 instead of 0.59 and 0.79 when brown pigments are not considered (Table 4). In the following, the discussion will therefore concentrate on the PROSPECT versions that include the brown pigments.

**Fig. 8:**
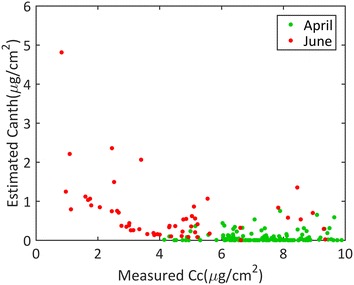
Scatter plots between estimated anthocyanins and measured carotenoid from PROSPECT PDb (PROSPECT D considering the brown pigment content). Green and red points correspond to measurements achieved at two nodes (April) and grain filling (June) stages respectively

When distinguishing between chlorophyll and carotenoids using the P5b and PDb versions, the estimated chlorophyll content was strongly correlated with the destructive measurements with similar performances as those observed when chlorophyll and carotenoids were pooled together (Table 4). Conversely, carotenoids were poorly estimated although PDb performed much better than P5b. A clear separation was observed between the April measurements corresponding to the greener leaves with more chlorophyllian pigments and the June measurements with overall lower values for all the PROSPECT versions (illustrated for PDb in Fig. 6a, b). Although PDb accounts for the anthocyanin pigments ($C_{anth}$), the corresponding estimates were very low with $C_{anth}$ < 0.5 µg/cm^2^ (Fig. 8) while larger values (0.5 µg/cm^2^ < $C_{anth}$ < 5 µg/cm^2^) were observed for the senescent leaves when the carotenoid (and thus chlorophyll) content was very low. For senescent leaves, the estimated anthocyanin content appeared to be correlated with the estimated carotenoid (and chlorophyll) pigment content (Fig. 8) although such a correlation was not reported from measured contents in previous studies [65]. This may be due to possible compensations between brown pigment, carotenoid and chlorophyll contents during the PROSPECT inversion process. In any case, results showed that accounting for the anthocyanin pigments for wheat leaves was not mandatory since these pigments were generally present only in very small quantities and anthocyanins present relatively weak absorption features. Thus, it appeared more efficient to estimate the content of pooled chlorophyllian pigments, $C_{abc}$, without considering the anthocyanin for wheat leaves. Furthermore, after bias correction all the PROSPECT versions performed similarly for $C_{abc}$ estimation in wheat leaves (Tables 4, 5).

However, absolute estimates of $C_{\text{ab}}$ and $C_{\text{abc}}$ from the PROSPECT model were significantly biased when compared to destructive measurements. Indeed, the specific absorption coefficients of the PROSPECT models were calibrated over a large range of species, including dicotyledonous and monocotyledonous leaves. The structure of dicotyledonous leaves is characterized by a well-developed spongy mesophyll that increases the average optical path while monocotyledonous leaves such as wheat have only a palisadic parenchyma where chloroplasts are concentrated [37]. Therefore, the values of the PROSPECT specific absorption coefficient calibrated over a large range of species might not represent accurately the actual individual values for each species. The distribution of the chlorophyllian pigments (e.g. pigment clumping) may also explain the different bias observed (Fig. 8) for yellow (June) and green leaves (April). For high values of chlorophyll content, e.g. green leaves, chlorophyll is concentrated within the chloroplasts and the chloroplasts themselves are organized in a clumped way in the cells. Conversely, the distribution of chlorophyllian pigments within yellow leaves is more uniform. As the specific absorption coefficients of PROSPECT were calibrated mostly over medium to high values of chlorophyll content, the estimated $C_{\text{abc}}$ for yellow leaves led to over-estimate the chlorophyll content due to the lower reflectance value expected for uniform pigment distribution as compared to a clumped situation.

All the PROSPECT versions provided very precise estimates of leaf water content (Tables 4, 5). This is mainly explained by the strong and specific absorption features of water. Conversely to what was observed for the chlorophyllian pigments, including the brown pigments did not improve the fitting process: actually, brown pigments are mainly absorbing in the visible domain where water shows only marginal absorption features. The bias observed between estimated and measured values of $C_{\text{w}}$, although significant, was much lower than that observed previously for $C_{\text{abc}}$. Some differences were noticed between PROSPECT versions, with P3 providing the lowest bias (Table 4). The smaller bias observed for $C_{\text{w}}$ as compared to that of $C_{\text{abc}}$ is mainly explained by the more even distribution of water within the leaf volume as compared to chlorophyll. Furthermore, the relative rRMSE (= 6%) obtained after bias correction was much lower than the one observed for $C_{\text{abc}}$ (rRMSE = 20%). This may be explained by the errors associated to the destructive measurements. For water content, the measurements were relatively accurate and precise because only few simple steps are required: measurements of the area and the fresh and dry weights. Conversely, the accuracy and precision associated to pigment content were expected to be degraded because of the several additional steps needed (leaf storage in the cold, extraction in a solution, spectrophotometer calibration…). Nevertheless, errors were also associated to the reflectance measurements, including the stability of the light source and that of the spectrophotometer, the characterization of the white references and the spectrophotometer calibration. Additional investigation should thus be conducted to quantify the repeatability of the destructive measurements as compared to the proposed method based on reflectance measurements. Furthermore, the interest of using a white background should also be investigated.

All the PROSPECT versions showed no correlation between the estimated $C_{m}$ and the corresponding destructive measurements with a systematic underestimation. However, the RMSE values were of the same order as those reported in previous studies generally conducted over larger range of $C_{m}$ values based on a similar inversion process (Table 6). However, the relative RMSE (rRMSE) was larger than for the other studies [26, 27, 46, 66–68]. Those latter, conversely to the present study, consider a large range of species where broadleaf and coniferous trees were often mixed with herbaceous plants. Therefore, the poor correlation observed was mainly explained by the very small variability of $C_{m}$ measured in this wheat experiment. Existing modified PROSPECT inversion methods that include the design of a specific merit function for $C_{m}$ [67] or the use of prior information [55, 68] and provided improved results over mixed trees could also be tested for $C_{m}$ estimation in wheat experiment in the future.

**Table 6:** Minimum, maximum of observed dry matter content, corresponding RMSE and relative RMSE (rRMSE) values of estimates from PROSPECT model inversion as reported in previous studies

Data set	Reference	PROSPECT versions	Reflectance/transmittance	Species	Min (mg/cm^2^)	Max (mg/cm^2^)	RMSE (mg/cm^2^)	rRMSE
Baret and Fourty (1997)	[26]	P3	Reflectance + transmittance	Temperate species and crops	2.2	8.3	1.4–1.6	0.23–0.26
Feret et al. (2008): LOPEX (Hosgood et al. 1994) ANGERS (Feret 2008) HAWAII (Feret 2008)	[46, 66]	P4, P5	Reflectance + transmittance	Temperate	1.7	15.2	3.5	0.26
				Temperate	1.7	33.1	2.6	0.08
				Tropical	6.4	22.9	4.9	0.30
Feret et al. (2011)	[27]	P5	Reflectance + (transmittance)^**^	Temperate and Tropical	0.8	33.1	3.1	0.09
Li and Wang (2011)	[67]	P4	Reflectance	Temperate species	2.6	11.9	2.7*	0.29
Ali et al. (2016)	[68]	P4	Reflectance + transmittance	Broadleaf	3.4	13.6	3.7*	0.36
				Conifer	1.1	29.1	8.6*	0.31
Present study		P3, P4, P5, PD	Reflectance	Wheat	4.0	6.0	2.5–3.1	1.25–1.55

* Indicates that better performances were obtained by modified PROSPECT model inversion methods

** Transmittance was not available for part of the data

### Comparison between VI and PROSPECT based methods for $C_{\text{abc}}$ and $C_{\text{w}}$ estimates

The ranking capacity between cultivars appeared to be very similar using either the VI or the PROSPECT based methods. It should be noticed that ranking did not require any calibration for VIs or bias correction for PROSPECT model inversion. However, in the context of phenotyping, the ranking between genotypes is not always sufficient. Estimates of the absolute values of the biochemical contents will allow using crop models to access functional traits. The results showed that biases were observed for estimates from PROSPECT inversion. This problem could be solved properly at least in two different ways: (1) by recalibrating the specific absorption coefficients for wheat leaves; (2) by changing the formalism of PROSPECT and including heterogeneous distribution of absorbers in the leaf. This will require a recalibration of the specific absorption coefficients over a large range of leaf types. Because of the limited amount of data available, as well as the fact that the only measured optical property was the reflectance over a white background, we did not perform a recalibration of the PROSPECT specific absorption coefficients. Therefore, a simple empirical recalibration of the raw estimates of PROSPECT using the destructive measurements was proposed. Results show that the performances of the vegetation indices were comparable to those of PROSPECT after this bias correction (Table 5). However, the PROSPECT model had the capacity to account for the effect of variation in the leaf surface and leaf mesophyll structure. Even though the relationship between $C_{m}$ and the leaf mesophyll structure was reported in previous studies [41, 69] when considering mixed species including both monocotyledons and dicotyledons, this relationship might not be so strong for a single species like wheat. Therefore, this may be important in the context of phenotyping experiments where new genotypes with particular surface or mesophyll features may be encountered.

## Conclusion

The ability of the PROSPECT model and vegetation indices to estimate wheat leaf biochemical content was evaluated. Reflectance measurements were collected over detached leaves using a spectrophotometer equipped with an integrating sphere. Leaves were put over a white Teflon background to enhance the absorption features and the PROSPECT model was adapted to account for this specific measurement configuration. Estimates from the inversion of several PROSPECT model versions were compared with destructive measurements. The considered versions differed by the explicit description of the absorption of some pigments (chlorophyll ab, carotenoid, anthocyanin, brown pigments) and the dataset used to calibrate the corresponding specific absorption coefficients and the refractive index. Results demonstrated that all the PROSPECT versions provided reasonable estimates of water and chlorophyll contents when the brown pigment content was used as an additional variable. This was particularly important when considering senescing leaves. Consideration of the anthocyanin did not offer major interest since wheat leaves did not show high values of anthocyanin content. The separation between chlorophyll and carotenoid contents did not bring significant improvement since they are strongly correlated. Consequently, the pooled chlorophyllian pigments (chlorophyll + carotenoids) should be used as a leaf trait. However, significant bias was observed for chlorophyllian pigments, probably due to the non-even distribution of chlorophyll in the leaf volume as well as some possible clumping of the chlorophyll pigments. Water content was estimated with a smaller bias, in relation to the more even distribution of the water in the leaf volume. In contrast with most of other studies involving the PROSPECT model applied to a large mix of species, this study concentrated on a single species. This highlights the limits of a generic formalism and calibration of the current PROSPECT models. Further investigations should therefore focus on a better description of the chlorophyll distribution in the leaf volume to account for differences between species. Furthermore, the bias and discrepancies observed in this study might be also partly explained by the measurement uncertainties associated to reflectance and biochemical contents.

PROSPECT estimates of chlorophyllian pigments and water contents were compared with empirical relationships based on vegetation indices. Results showed very similar performances in terms of ranking as well as in terms of RMSE after bias correction for PROSPECT model estimates. Although VIs provided a very simple and straightforward method for biochemical content estimates, PROSPECT model inversion offered the advantage to explicitly account for genotypic differences in leaf surface features, $R_{surf}$ and mesophyll structure ($N$). However, these two additional variables should be more deeply investigated to evaluate their interest as potential new traits. Indeed, $R_{surf}$ could allow characterizing the glaucosity observed between genotypes and conditions through the differences in leaf ‘color’ due to leaf surface features.

Finally, this study indicates that non-destructive methods may provide similar or better precision of chlorophyllian pigments and water contents as compared to classical destructive measurements [29]. However, the repeatability of these traits should be more formally compared over a large phenotyping dataset. The currently limited throughput of the indirect methods based on leaf reflectance achieved in the lab may be replaced in the close future by the development of new imaging techniques achieved at the canopy level as suggested by [70].

## Additional file

**Additional file 1.**
Contains the experimental dataset used in this study e.g. the measured wheat leaf reflectance over the white background and the corresponding chlorophyll a and b, carotenoid, dry matter and water contents. The EXCEL file includes 4 sheets. The first sheet is the description of the dataset. The sheet ‘reflectance_Chl’ includes the leaf reflectance over the white background measured from 400 mm to 2200 mm for 186 wheat leaves and the corresponding chlorophyll a and b and carotenoid content measured destructively. The sheet ‘reflectance_Cw_Cm’ is the leaf reflectance over the white background measured from 350 mm to 2500 mm for 186 wheat leaves and the corresponding water and dry matter contents from destructive measurements. Sheet ‘reflectance of white background’ describes the reflectance of the white background.

## Abbreviations

$$B_{max}$$: maximum bound
$$B_{min}$$: minimum bound
$C_{ab}$: chlorophyll a and b content (µg/cm^2^)
$C_{c}$: carotenoid content (µg/cm^2^)
$C_{abc}$: chlorophyll and carotenoid content (µg/cm^2^). $C_{abc} = C_{ab} + C_{c}$
$C_{Anth}$: anthocyanin content (µg/cm^2^)
$C_{m}$: dry matter content (mg/cm^2^)
$C_{w}$: water content (mg/cm^2^)
$C_{bp}$: brown pigment content (no unit)
N: mesophyll structure index (no unit)
$R_{surf}$: reflectivity of the leaf surface (no unit)
$J$: cost function (no unit)
$f_{wb}$: fraction of background illuminated by the incident light in the integrating sphere (no unit)
$R_{leaf}$: reflectance of the leaf (over black background) (no unit)
$T_{leaf}$: transmittance of the leaf (no unit)
$R_{leaf}^{wb}$: reflectance of the leaf over the white background (no unit)
$R_{vol}^{wb}$: reflectance of the leaf without the epidermis over the white background (no unit)
$R_{wb}$: reflectance of the white background (no unit)
$M_{fresh}$: leaf fresh weight (g)
$M_{fresh}$: leaf dry weight (g)
$S$: leaf area (cm^2^)
$DHRF$: directional hemispherical reflectance (no unit)
$n$: refractive index (no unit)
RMSE: root mean square error
